# Next-generation sequencing study on poorly differentiated carcinoma derived from a thirty-year-old epidermoid cyst: A case report

**DOI:** 10.3389/fonc.2023.1017624

**Published:** 2023-04-03

**Authors:** Woosuk Choi, Joseph Kyu-hyung Park, Seung Geun Song, Baek-kyu Kim

**Affiliations:** ^1^ Department of Plastic and Reconstructive Surgery, Seoul National University College of Medicine, Seoul National University Bundang Hospital, Seoul, Republic of Korea; ^2^ Department of Pathology, Seoul National University College of Medicine, Seoul, Republic of Korea

**Keywords:** epidermoid cyst, squamous epithelium, next-generation sequencing, DNA copy number variation, malignant transformation

## Abstract

Although epidermoid cysts are frequently seen as benign lesions, they are highly uncommon to develop into cancerous lesions. A 36-year-old man with a cystic mass present on his left flank since childhood presented to our department. Based on the patient’s medical history and abdominal computed tomography scan, we excised the lesion under the suspicion of an epidermoid cyst. Histopathological evaluation revealed the presence of poorly differentiated carcinoma with squamoid and basaloid differentiation, which showed a strong possibility of carcinoma arising from an epidermal cyst. Next-generation sequencing using TruSight oncology 500 assay showed copy number variation of *ATM* and *CHEK1* genes.

## Introduction

Epidermoid cysts are the most common benign skin lesion with a high prevalence, compromising about 85-90% of all excised subcutaneous cysts (1). However, malignant transformations of epidermoid cysts have been rarely reported, with the transformation rate to squamous cell carcinoma (SCC) from 0.011 to 0.045% (2). Due to the rarity of the phenomenon, some cases of malignant transformationa have been reported in the skin, the abdominal cavity, and the intracranial space; however, the etiology or the risk factors are not well known yet (3, 4). We report a case of poorly differentiated carcinoma with squamoid and basaloid differentiation arising from a suspected epidermoid cyst, diagnosed using immunohistochemistry and next-generation sequencing using TruSight™ Oncology 500 (TSO500). This study was approved beforehand by the Institutional Review Board of Seoul National University Bundang Hospital (IRB No. B-2208-773-702) and performed in accordance with the principles of the Declaration of Helsinki. All patients provided written informed consent for publication and use of their images.

## Case report

**Figure 1:**
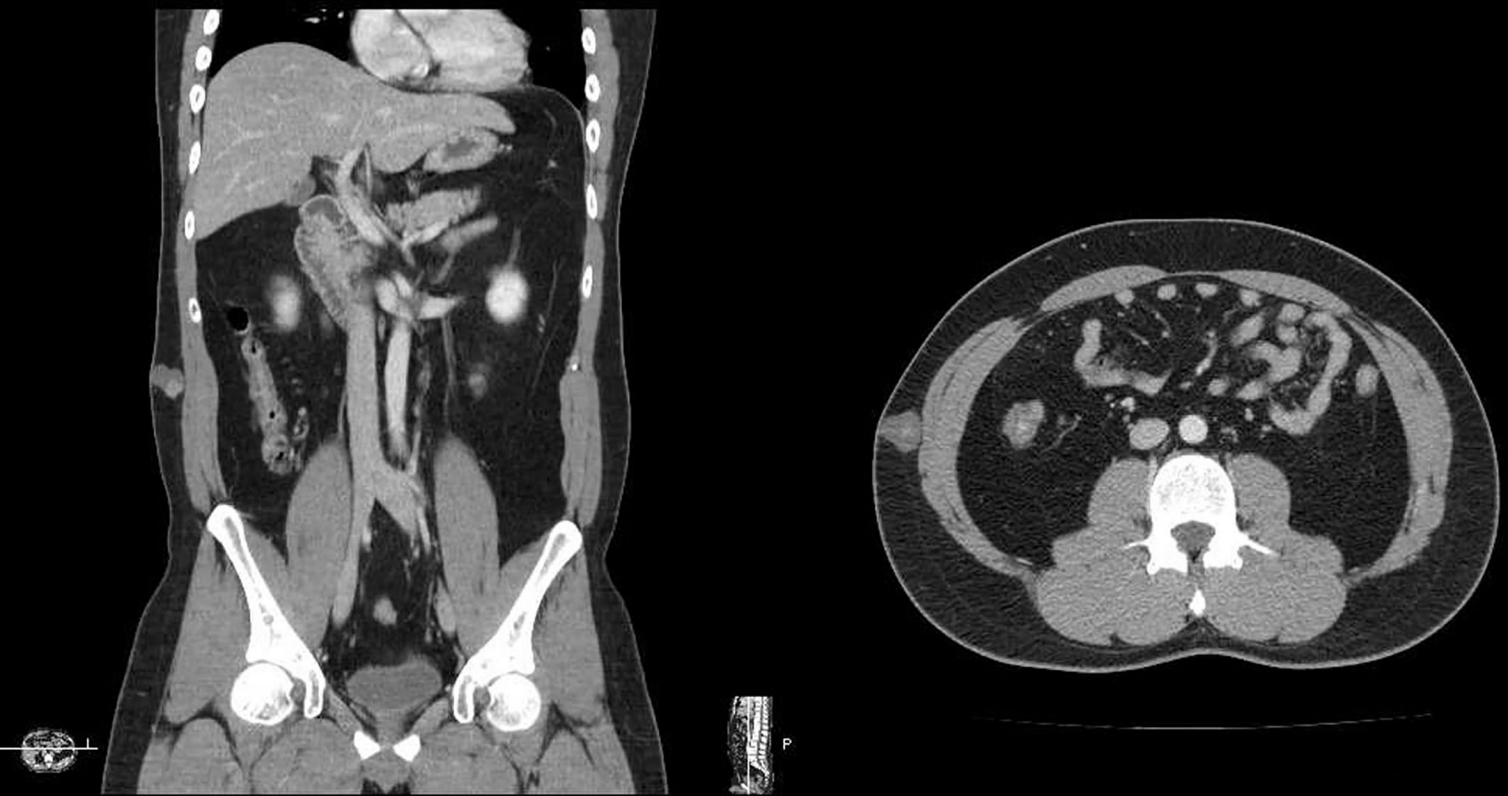
A 2.2 cm cystic lesion at the right flank isolated in the subcutaneous fat layer.

A 36-year-old male visited our clinic with a flank mass that has existed since childhood and has rapidly grown in size over six months. On the computed tomography (CT) scan, a 2.2cm cystic mass in the subcutaneous tissue was observed (**Figure 1**). An excisional biopsy was performed under the initial clinical impression of epidermoid cyst. Following the pathological diagnosis of malignancy, further wide excision was performed with a 2cm margin from the previous scar and deep to the deep fascia of the external oblique muscle to obtain clear surgical margins.

**Figure 2:**
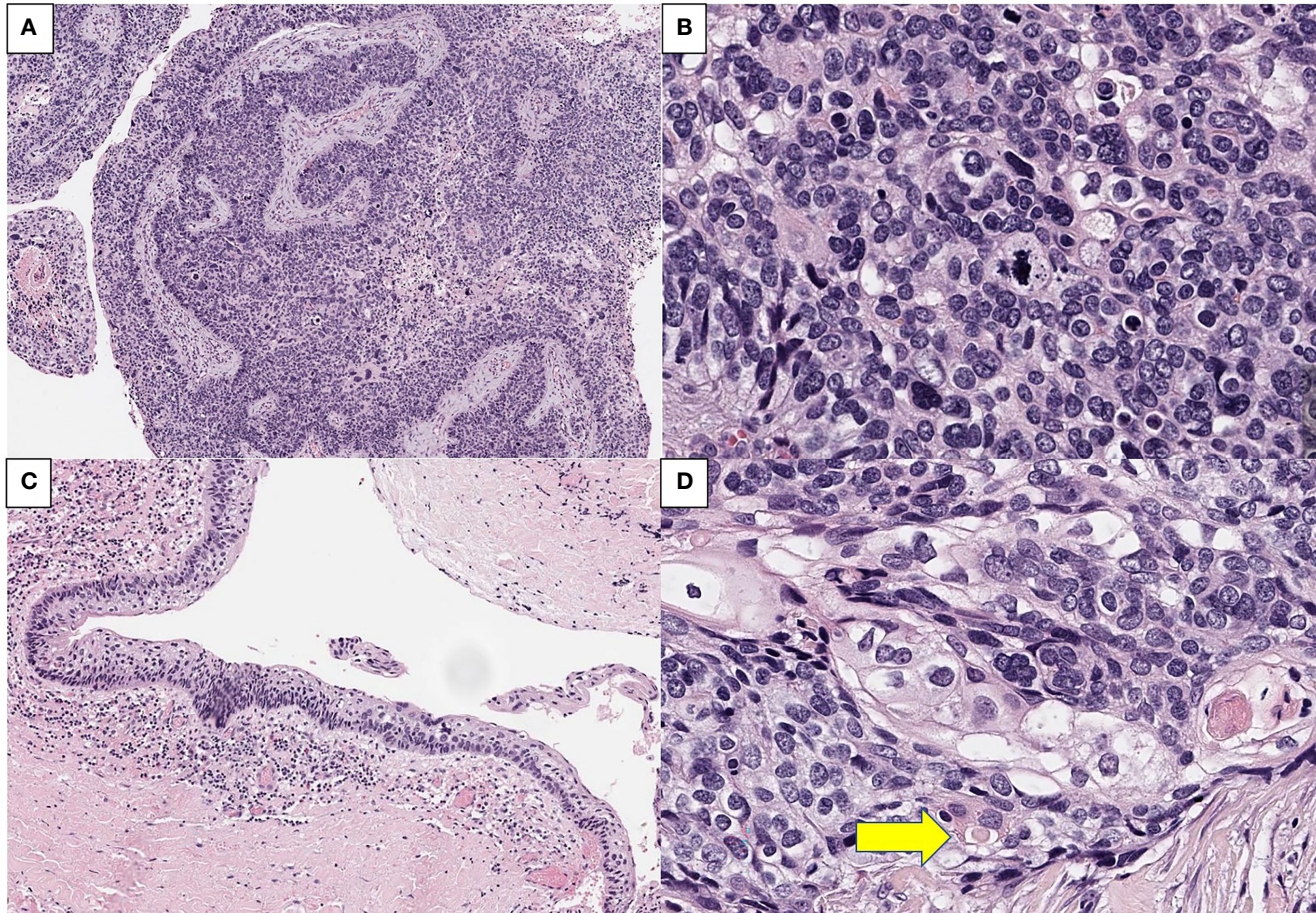
Representative H&E images of the tumor. **(A)** An infiltrative tumor with connection to cystic lining with high grade squamous intraepithelial lesion (x 100). **(B)** the tumor cells showed severe nuclear atypia and clear, vacuolar cytoplasm (x 800). **(C)** adjacent squamous cell carcinoma *in situ* lesion with no direct connection to normal skin epithelium (x 200). **(D)** cells with clear squamous differentiation and keratin pearl (arrow, x800).

**Figure 3:**
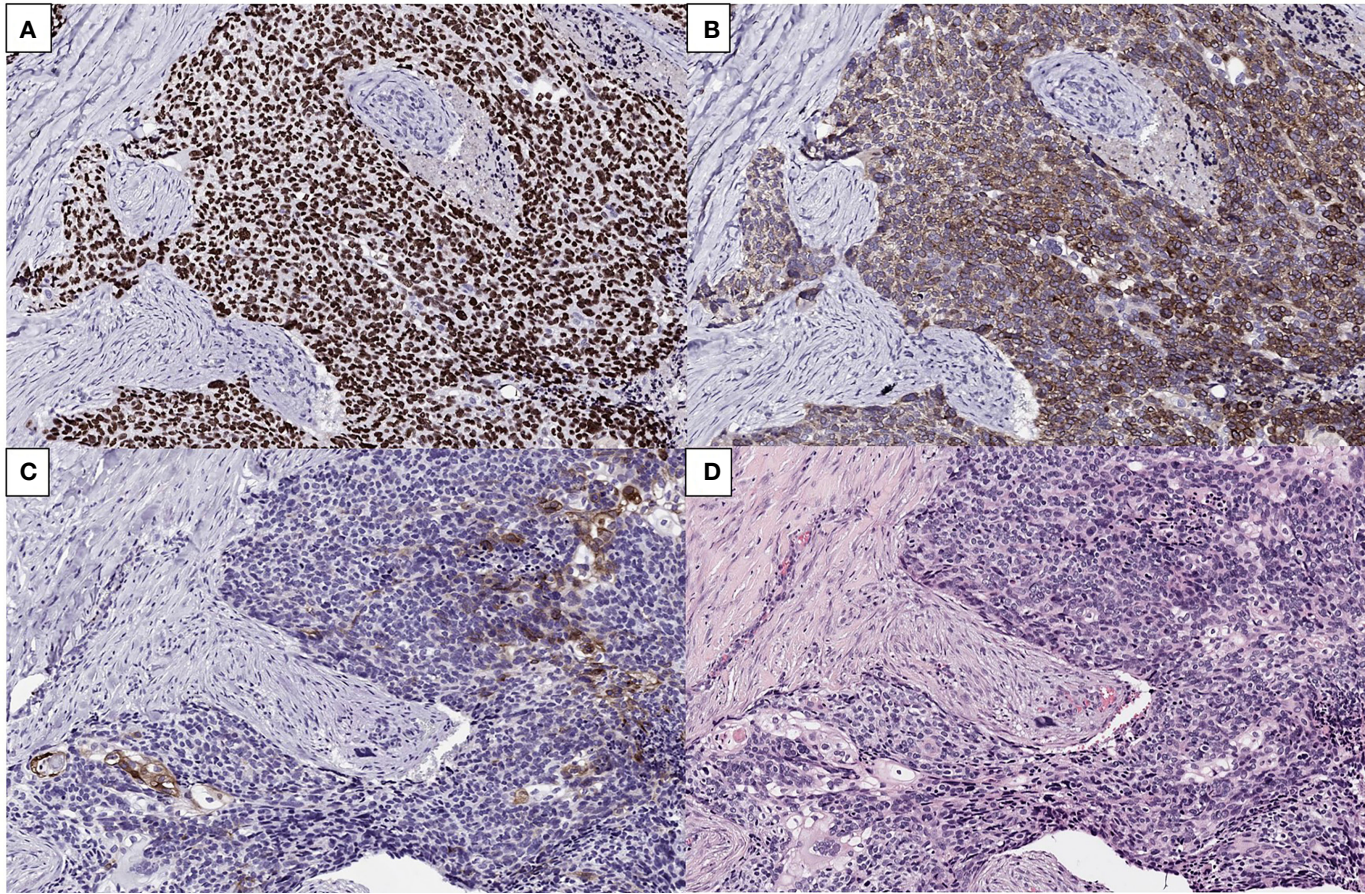
**(A, B)** Immunohistochemistry showing diffuse strong positivity of the tumor cells for p63 **(A)** and bcl-2 protein **(B)** (×200). **(C, D)** EMA stain revealing focal positivity in well differentiated cells **(C)** and corresponding H&E image **(D)** (x200).

Microscopically, a malignant lesion with an infiltrative margin was observed. The cells had a high nuclear to cytoplasm ratio and often contained clear vacuoles in their cytoplasm. The nuclei showed severe nuclear atypia and partial basaloid features (**Figures 2A, B**). A lesion corresponding to squamous cell carcinoma *in situ* was also observed at the edge of the main lesion, but no connection with normal skin epithelium was observed (**Figure 2C**). In some areas, cells with clear squamous differentiation were observed and cells containing keratin pearl were also noted (**Figure 2D**). In immunohistochemical staining, the cells of the lesion showed diffuse strong positivity for p63 and bcl-2 while epithelial membrane antigen (EMA) was positive only in well differentiated squamous cells (**Figure 3**). Taken together, the pathological diagnosis was poorly differentiated carcinoma with squamous and basal differentiation.

As the tumor was situated in the subcutaneous tissue and no direct connection to overlying skin was observed on gross and histological findings, metastatic carcinoma could not be ruled out. Further systemic evaluation including whole-body fluorodeoxyglucose-positron emission tomography (FDG-PET), bronchoscopy, esophagogastroduodenoscopy, and colonoscopy did not reveal any other cancerous lesions. Also, since the flank mass was present since childhood, the possibility of the tumor being a metastatic lesion was very low. Considering the histopathological features, clinical examination results and past medical history, the final diagnosis was likely to be primary poorly differentiated carcinoma with squamous and basal cell differentiation arising from an epidermoid cyst.

Since TNM staging system is not available for the tumor, NGS using TSO500 assay was performed to determine if additional postoperative treatments such as adjuvant chemotherapy or immunotherapy were applicable (**Supplementary Table 1**). There was no detected translocation or genomic alteration on Tier I and II genes, but gene fold changes were identified as copy number variation in homologous recombination-related genes *ATM* and *CHEK1*. The total tumor mutation burden was 5.5/MB (**Table 1**). No additional postoperative treatments were performed. No recurrence or metastasis was observed until the two-year follow-up visit.

**Table 1:** TruSight Oncology 500 assay result (Tier I and II only).

SNV/INDEL (Allele Frequency ≥ 2%)		No detected SNV/Indel in Tier I and II genes					
Fusion and splice variants (RNA)	Fusion (Fusion supporting Reads ≥ 3)	No detected translocation in Tier I and II genes					
	Splice variants	No detected translocation in Tier I and II genes					
Copy Number Alteration (Fold change ≥ 2.2)		No detected CNV in Tier I and II genes					
Homologous Recombination-related Genes	SNV/INDEL† (Allele Frequency ≥ 2% with pathogenic mutation, ≥ 25% with truncation mutation)	No detected genomic alteration in 25 homologous recombination-related genes included in *SNUBH pancancer TruSight Oncology 500 LVII					
	CNV‡ (Fold change ≤ 0.8)	Gene	Type	Locus	Fold change	Estimated copy number (purity-adjusted)	Tier
		ATM	LOSS	11q22.3	0.39	0.257	III
		CHEK1	LOSS	11q24.2	0.41	0.314	III
Microsatellite instability (MSI-H ≥ 20% unstable loci)		Unstable Loci	Passing Loci	Present Unstable MSI sites		Prediction of MSI Status	
		2	124	2		MSS/MSI-L	
Tumor Mutation Burden (N(var) x 10^6/ExonLength)		Number of Passing Eligible Variants		Coding Region Size in Megabases		Total TMB	
		7		1.27		5.5/MB	

*SNUBH pancancer TruSight Oncology 500 LVII (ATM, ATR, BAP1, BARD1, BLM, BRCA1, BRCA2, BRIP1, CDK12, CHEK1, CHEK2, FANCL, MRE11A, NBN, PALB2, POLD1, RAD50, RAD51, RAD51B, RAD51C, RAD51D, RAD52, RAD54L, PPP2R2A, XRCC2).

## Discussion

Epidermoid cysts, commonly seen as benign intradermal or subcutaneous tumors, are defined as cutaneous cystic mass appearing on the whole body with epidermis-like epithelial walls (5). Though epidermoid cysts grow slowly and are mostly benign, malignant transformations have rarely been reported. Possible malignant tumors that can develop from epidermoid cysts include SCC, basal cell carcinoma, Paget’s disease, Bowen’s disease, mycosis fungoides, Merkel cell carcinoma, and malignant melanoma. Locations of previously reported malignant transformations include the skin, the intracranial, and the abdominal cavity (3, 4). SCC is the most commonly reported transformation among the abovementioned malignancies, with a rate of 0.011%–0.045% (2, 6).. A cohort study of 41 cases from 1976 to 2018 showed that the most common site of SCC transformation was head and neck (54.8%), and the mean time from occurrence to diagnosis was 92.6 months (7).

The etiology of malignant transformation of the epidermoid cyst has not yet been determined. The risk factors for cutaneous SCC, such as radiation, immunosuppression, scars, and chronic inflammation, are also considered risk factors for this transformation (6). Clinically, pain, fast growth, overlaying skin abnormalities, and continuous drainage are some warning signs of neoplastic progression (7).

In the present case, TSO500 assay was performed to characterize the tumor and assist in determining the need for adjuvant therapy. At our institution, SNV/Indel, fusion or splice variants (RNA), copy number variation(CNV), twenty-five homologous recombination-related genes, microsatellite instability, and tumor mutation burden (TMB) are reported. Interestingly, CNV with fold change below 0.8 of *ATM* and *CHEK1* homologous recombination-related genes were identified in our case.

The activation of ATR-Chk1 and ATM-Chk2 pathways are essential in DNA damage response and cell cycle checkpoints. The ATR-Chk1 pathway, in particular, manages a broad spectrum of DNA abnormalities (8, 9). Furthermore, ATM loss at the cellular level is associated with chromosomal instability and radiosensitivity (10). The activation of ATM and downstream checkpoint kinases Chk1 has a major role in eradicating precancerous cells. The mutations identified in the present case may have promoted tumor growth and cell proliferation (11–13).

Both ATM gene and CHEK1 gene mutations have been found to be related to various cancers through previous studies. ATM gene is one of the most frequently aberrant genes in sporadic cancers, especially hematologic malignancies, according to next-generation sequencing studies (14). Numerous ATM mutations have been identified and linked to a moderate risk of BC occurrence (15). In the case of CHEK1 gene, the association with endometrial and colorectal cancer is known (16), and the association with solid tumor is also being studied (17).

To the best of our knowledge, this is the first report of NGS on carcinoma originating from an epidermoid cyst. Due to the low incidence of such malignant transformations, the mechanism behind it has been difficult to research. With the increased availability of NGS and other sequencing modalities, more data can be collected to elucidate our understanding of these malignant transformations and to assist in finding new adjuvant immunotherapies for these entities.

## Data availability statement

The datasets presented in this study can be found in online repositories. The names of the repository/repositories and accession number(s) can be found in the article/**Supplementary Material**.

## Ethics statement

Written informed consent was obtained from the individual(s) for the publication of any potentially identifiable images or data included in this article.

## Author contributions

WC, JP, and BK contributed to conception and design of the study. WC wrote the first draft of the manuscript. WC and JP wrote sections of the manuscript. SS selected proper pathologic image and interpreting those images. All authors contributed to the article and approved the submitted version.
