# Supplementary material for: Next-generation sequencing study on poorly differentiated carcinoma derived from a thirty-year-old epidermoid cyst: A case report
Source: Front Oncol. 2023 Apr 3;13:1017624. doi: 10.3389/fonc.2023.1017624 (PMC10106615; doi:10.3389/fonc.2023.1017624)
Supplement: Supplementary file 1 [file Table_1.docx]

| **Supplementary Table 1.** SNUBH pancancer TruSight Oncology 500 LVII | | | | |
| --- | --- | --- | --- | --- |
|  |  |  |  |  |
| **Test and Database Version information** | | |  |  |
| Sequencing Platform | Type of Sequencers | | | NextSeq 550Dx |
|  | Library Preparation methods | | | Target Enrichment DNA and RNA Workflow |
|  | Target capture methods | | | Biotinylated probes  OPD2 (Oncology Probes DNA 2) for DNA library  OPR1 (Oncology Probes RNA 1) for RNA library |
|  | Read Type | | | Paired ends |
|  | Read length | | | 200bp |
| Analysis Platform Information | Reference Genome | | | GRCh37/hg19 |
|  | Panel | | | SNUBH pancancer-TruSight Oncology 500 LVII |
|  | Quality check & trimming | | | TruSight Oncology 500 v2.1 Local App |
|  | Alignment tools | | | TruSight Oncology 500 v2.1 Local App  (BWA, ReCo, Gemini, STAR) |
|  | Variant calling tools | | | TruSight Oncology 500 v2.1 Local App  (CRAFT, Pisces, Pepe2, Scylla, Nirvana, TMB raider, Hubble, SpliceGirl, Manta) |
|  | Chromosome coordinate system | | | 1-based |
|  | Annotation tools and databases | | | tools - SnpEff v4.3, SnpSift v4.3  databases - dbNSFP v4.1, 1000 genome phase 3, ExAC v0.3, gnomAD exome 2.1, dbSNP 151, ESP6500SI-V2, clinvar 20200609 |
|  |  |  |  |  |
|  |  |  |  |  |
| **Homologous Recombination-related Genes** | | | |  |
| SNV/INDEL | VAF ≥ 2% with Pathogenic or Likely Pathogenic or Drug Response   ≥ 25% with Frameshift or Stopgain | | | |
| CNV | Fold change ≤ 0.8 | | | |
|  |  |  |  |  |
| For SNV/INDEL, the HRD is the result of analysis for 25 genes, including ATM, ATR, BAP1, BARD1, BLM, BRCA1, BRCA2, BRIP1, CDK12, CHEK1, CHEK2, FANCL, MRE11A, NBN, PALB2, POLD1, RAD50, RAD51, RAD51B, RAD51C, RAD51D, RAD52, RAD54L, PPP2R2A, XRCC2. | | | | |
| For CNV, the Fold change less than or equal to 0.8 is reported for 5 genes, ATM, BRCA1, BRCA2, CHEK1, CHEK2. | | | | |
|  |  |  |  |  |
| **Microsatellite Instability** | |  |  |  |
| The MSI is the calculated ratio of unstable sites over QC passed regions of the predefined ones of MSI related sites. | | | | |
|  |  |  |  |  |
| **Tumor Mutation Burden** | |  |  |  |
| TMB = Eligible Variants / Effective panel size | | | |  |
| Eligible Variants | (1) Variant with an observed allele count ≥ 10 in databases excluded | | | |
|  | 2) Grouped variants ≥ 5 variants on the same chromosome of removed variant by first strategy  with similar frequency excluded | | | |
|  | (3) Variants in the coding region (RefSeq Cds) | | | |
|  | (4) 5% ≤ Variant Frequency < 90% | | | |
|  | (5) Coverage ≥ 50X | | | |
|  | (6) SNVs and Indels (MNVs excluded) | | | |
|  | (7) Nonsynonymous and synonymous variants | | | |
|  | (8) Variants with COSMIC count ≥ 50 excluded | | | |
| Effective Panel Size | (1) Total coding region with coverage > 50X | | | |
|  | (2) Excluding low confidence regions in which variants are not called | | | |
